# AMSC‐sEVs Ameliorated Crohn's Disease by Inhibiting Macrophage‐Myofibroblast Transition Through the Delivery of MFGE8

**DOI:** 10.1111/cpr.70159

**Published:** 2026-01-02

**Authors:** Minghao Xie, Qiang Liu, Zhizhong Xiong, Jian Li, Ruiri Jin, Lei Lian, Zhengrong Li

**Affiliations:** ^1^ Department of General Surgery The First Affiliated Hospital, Jiangxi Medical College, Nanchang University Nanchang Jiangxi China; ^2^ Department of Gastrointestinal Surgery The Sixth Affiliated Hospital, Sun Yat‐sen University Guangzhou Guangdong China; ^3^ Department of Gastroenterology The First Affiliated Hospital, Jiangxi Medical College, Nanchang University Nanchang Jiangxi China

**Keywords:** Crohn's disease, macrophage‐myofibroblast transition, macrophages, MFGE8, Tregs

## Abstract

This study elucidates the critical role of macrophage‐myofibroblast transition (MMT) in the pathogenesis of intestinal fibrosis in Crohn's disease (CD). Through analysis of stricturing intestinal tissues from CD patients and TNBS‐induced CD mouse models, we demonstrated that TGF‐β1 activates the MAPK signalling pathway to induce MMT in macrophages (Mø), resulting in increased expression of α‐SMA and collagen production. Importantly, these MMT‐derived myofibroblasts secrete CCL17, which recruits CCR4^+^ regulatory T cells (Tregs) to fibrotic lesions, creating a pro‐fibrotic microenvironment. Further investigation showed that the adoptive transfer of Mø exacerbated fibrosis in CD mice, whilst Mø depletion attenuated this process. Therapeutically, adipose‐derived mesenchymal stromal cells‐derived extracellular vesicles (AMSC‐sEVs) could effectively deliver MFGE8 to inhibit MAPK activation, thereby suppressing MMT and reducing CCL17‐mediated Treg recruitment. Treatment with AMSC‐sEVs significantly improved intestinal fibrosis in CD mice, as evidenced by reduced collagen deposition and improved histological scores, whereas MFGE8 knockdown in AMSC‐sEVs diminished these protective effects. These findings not only establish MMT as a key mechanism driving CD‐associated intestinal fibrosis through the CCL17‐CCR4 axis but also highlight AMSC‐sEVs as a promising cell‐free therapeutic strategy targeting this pathological process.

## Graphical abstract

Our study demonstrated that TGF‐β1 activated MAPK signalling pathway and promoted the MMT and secretion of CCL17 under CD‐associated intestinal inflammatory conditions. AMSC‐sEVs can deliver MFGE8, which inhibits MMT and CCL17‐mediated Treg recruitment by suppressing the MAPK signalling pathway, thereby alleviating intestinal fibrosis.

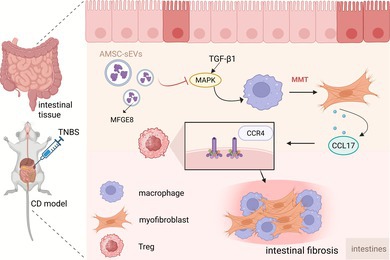

## 1 Introduction

Crohn's disease (CD), a chronic inflammatory bowel disease (IBD) of the gastrointestinal tract characterised by recurrent transmural inflammation, has a pathological process often accompanied by severe symptoms, such as intestinal stenosis and obstruction, which significantly affect the quality of life of patients [1]. In recent years, with the gradual revelation of the multifactorial interactions of genetic susceptibility, environmental factors, gut microbiota dysbiosis and defective intestinal barrier function, research on the pathogenesis of CD has made significant progress [2]. However, despite improvements in treatments, intestinal fibrosis remains a major cause of intestinal stenosis and intestinal obstruction in patients with CD, and the central mechanism involves the abnormal deposition of extracellular matrix (ECM) triggered by excessive activation of intestinal muscle fibroblasts [3]. Therefore, an in‐depth investigation of the key cellular events involved in the intestinal fibrosis process is crucial for the development of effective therapeutic strategies against CD.

Several studies have confirmed that the MMT plays a key role in diseases related to fibrosis [4]. As important effector cells of the innate immune system, Mø undergo phenotypic transformation in response to inflammatory stimuli and acquire myofibroblastic properties, inducing overproduction of ECM and secretion of pro‐fibrotic factors, accelerating the fibrotic process [5]. MMT has been widely reported in diseases such as renal fibrosis and pulmonary fibrosis, but its specific mechanism of action in CD‐related intestinal fibrosis is unclear [6, 7]. Notably, increased expression of Osteopontin (OPN) in Mø has been observed in a mouse model of colitis, a change that would further activate fibroblasts and lead to ECM deposition [8]. These findings suggest that MMT may play a key role in CD intestinal fibrosis.

Regulatory T cells (Tregs) are a subpopulation of CD4^+^ T cells with immunosuppressive functions, which play a key role in the repair of intestinal inflammation through the secretion of anti‐inflammatory factors such as interleukin 10 (IL‐10) and transforming growth factor beta (TGF‐β) [9]. However, with the continuation of inflammation, large amounts of TGF‐β secreted by Tregs activate the TGF‐β/Smad signalling pathway to promote fibrogenesis [10, 11]. Furthermore, it has been demonstrated that Mø can recruit Tregs to the injury site and promote fibrosis by secreting Chemokine (C‐C motif) Ligand 17 (CCL17), which binds to the Chemokine (C‐C motif) Receptor 4 (CCR4) receptor on Tregs [12]. However, whether MMT regulates Treg recruitment through the CCL17/CCR4 axis and its role in CD fibrosis is not yet clear. Some studies have shown that under stimulation of the cytokine TNF‐α/IFN‐γ, inhibition of MAPK and NF‐κB reduces the levels of CCL17 in stimulated human keratinocytes [13]. It indicates that activation of MAPK may contribute to the expression and secretion of CCL17. Currently, there is no evidence to suggest that the MMT activates the MAPK signalling pathway and induces the secretion of CCL17. Therefore, the MMT regulatory mechanism in MAPK and CCL17 remains to be further explored.

Extracellular vesicles are nanoscale vesicles secreted by cells that exhibit unique advantages in disease modulation [14]. In particular, small extracellular vesicles (AMSC‐sEVs) secreted by adipose‐derived mesenchymal stem cells are enriched with a variety of biologically active molecules, which can deliver RNA, miRNA, DNA and other substances to the target cells through paracrine secretion, thus regulating the function of the recipient cells [15]. Our previous study showed that AMSC‐sEVs ameliorate intestinal fibrosis by delivering Milk Fat Globule EGF Factor 8 (MFGE8) [16]. MFGE8 is a multifunctional protein capable of inhibiting a wide range of tissue fibrosis by modulating the TGF‐β/Smad3 signalling pathway [17, 18]. However, as of now, it is unclear whether MFGE8 alleviates CD‐associated intestinal fibrosis by inhibiting MMT.

In this work, we carried out in vivo and in vitro assays and demonstrated that TGF‐β1 activated MAPK signalling pathway and promoted the MMT and secretion of CCL17 under CD‐associated intestinal inflammatory conditions. CCL17 promoted intestinal fibrosis by binding to the receptor CCR4 on the surface of Tregs and recruiting Tregs to intestinal injury sites. AMSC‐sEVs inhibit MAPK signalling pathway through the delivery of MFGE8, thereby interfering with the MMT and ultimately ameliorating intestinal fibrosis.

## 2 Materials and Methods

### 2.1 Participants

CD patients were recruited from the Sixth Affiliated Hospital of Sun Yat‐sen University. IBD diagnosis was conducted following European Crohn's and Colitis Organisation guidelines [19]. Inclusion and exclusion criteria were made in accordance with the previous study described [20]. Stricturing intestinal tissue and adjacent normal intestinal tissue samples were obtained from CD patients after surgical resection. The specimens were subjected to fixation and embedding or frozen in liquid nitrogen, and used for histochemical analysis and flow cytometry analysis. All participants signed the informed consent. The protocols conformed to the Declaration of Helsinki and were approved by the ethical review committee of the Sixth Affiliated Hospital of Sun Yat‐sen University (2021ZSLYEC‐075).

### 2.2 Isolation and Identification of sEVs

AMSCs were isolated from inguinal and infrarenal adipose tissues, as a previous study described [21]. AMSCs were cultured in MSCs complete culture medium containing 2% EVs‐free foetal bovine serum (FBS; Thermo Fisher Scientific), and sEVs were from the supernatant of AMSCs. sEVs were isolated by differential ultracentrifugation. For removal of dead cells and cell debris, the supernatant was subjected to centrifugation at 2000 × *g* for 15 min and 16,000 × *g* for 45 min. Then, the supernatant was filtered through a 0.22‐μm filter. The filtered supernatant was centrifuged again at 120,000 × *g* for 70 min at 4°C for two times. The pellet was washed with PBS, and then resuspended in PBS for further use. The ultrastructure of sEVs was confirmed using a NanoSight NS300 (Malvern Instruments Ltd., Malvern, UK) and transmission electron microscope (TEM). The surface markers CD63 of sEVs were analysed by western blotting (WB).

### 2.3 Isolation and Differentiation of Mø

Mice were euthanized by cervical dislocation. Under aseptic conditions, bone marrow tissue was isolated from the femur and made into a single‐cell suspension. Cells were filtered through a sterile 400 mesh nylon sieve and centrifuged at 4°C, 1200 r/min for 4 min. After that, cells were treated with RBC Lysis Buffer (Thermo Fisher Scientific) at 4°C for 5 min. Cells were then cultured in DMEM containing 10% FBS at 37°C and 5% CO_2_ for 6 h, and then incubated with 25 ng/mL M‐CSF for 10 days. For differentiation of myofibroblasts, the collected cells (Mø) were treated with 15 ng/mL TGF‐β1 for 48 h or combined with 10 μM SB203580 for 30 min. Mø were treated with 5 μg/mL AMSC‐sEVs for 12 h.

### 2.4 Cell Transfection

Short hairpin RNA (shRNA) specially targeting MFGE8 (sh‐MFGE8‐1/2/3) was constructed for MFGE8 silencing. Scrambled shRNA served as control. Lentivirus‐mediated pGL3‐Luc (LV‐Luc) vector was used to label Mø. All vectors were obtained from Genepharm (Shanghai, China). AMSCs were transfected with sh‐MFGE8 or sh‐NC using Lipofectamine 2000 reagent. Mø were infected with LV‐Luc applying 0.5 μg/mL polybrene.

### 2.5 Extracellular Vesicle Cellular Uptake Analysis

To detect the phagocytosis of Mø on sEVs, the isolated sEVs were stained with PKH67 using the Green Fluorescent Cell Linker Kit (Sigma‐Aldrich, St. Louis, MO, USA) following the protocol of manufacturer. The labelled sEVs were incubated with Mø at 37°C for 6 h. The nuclei were counterstained with DAPI. Finally, the Mø were observed under the fluorescence microscope (Olympus, Tokyo, Japan).

### 2.6 Animals

Balb/c male mice (aged 4–5 weeks) were purchased from the Charles River Laboratories (Beijing, China) and bred in a SPF facility with constant temperature (20°C–24°C) and constant humidity (40%–60%). In the following reported experiments on live vertebrates and methods used, all relevant guidelines and regulations, particularly ARRIVE guidelines 2.0, have been fully considered and noted. The animal experiments were conducted according to state guidelines and approved by the Ethical Committee of the First Affiliated Hospital, Jiangxi Medical College, Nanchang University (CDYFY‐IACUC‐202305QR045).

### 2.7 CD Mouse Model

Mice were randomly divided into six groups: Control, Model, CL, CL + Mø, AMSC‐sEVs and AMSC‐sEVs‐shMFGE8. CD mouse model was constructed by increasing concentrations of 2,4,6‐trinitrobenzenesulfonic acid solution (TNBS) as previously studied [22]. In brief, mice were infused with 100 μL of TNBS (1.5 mg/100 μL TNBS for weeks 1 and 2, 2.0 mg/100 μL TNBS for weeks 3 and 4 and 2.5 mg/100 μL TNBS for weeks 5–7) into the colon every day for 7 weeks. Infusion was performed using a 4 cm stainless cannula attached to a 1 mL syringe. The mice were hung upside down for at least 1 min immediately after infusion to ensure sufficient contact of TNBS and the colon mucosal surface. For Mø depletion, mice were intraperitoneally injected with 200 μL of Clodronate liposomes (Mø scavenger, CL; FormuMax Scientific, CA, USA) twice weekly for weeks 3–7. In CL + Mø group, mice were intraperitoneally injected with 200 μL of CL + Mø twice weekly for weeks 3–7. In AMSC‐sEVs and AMSC‐sEVs‐shMFGE8 groups, mice were injected with 200 μg/200 μL AMSC‐sEVs or AMSC‐sEVs‐shMFGE8 into tail vein twice weekly for weeks 3–7. Normal mice were infused with 100 μL of PBS or injected with 200 μL PBS into tail vein as Control.

### 2.8 Assessment of Colon Damage

Body weight, stool features and faecal occult blood of mice were monitored every other day. The disease activity index (DAI) and colon morphological damage index (CMDI) score criteria are shown in Tables 1 and 2. Scores of two gross morphology values were averaged for statistical analysis.

**TABLE 1:** Scoring of disease activity index.

Weight loss (%)	Stool consistency	Occult/gross bleeding	Score
Normal	Normal	Normal	0
1–5			1
5–10	Loose stools	Hemoccult+	2
10–20			
> 20	Diarrhoea	Gross bleeding	4

*Note*: Normal stools = well‐formed pellets, loose stools = pasty and semiformed stools which do not stick to the anus and diarrhoea = liquid stools that stick to the anus.

**TABLE 2:** Colonic mucosal damage index (CMDI) scoring criteria.

Gross morphologies	Symptoms	Score
Correlation with the surrounding tissues during sampling	No adhesion	0
	Mild adhesion (colon and other tissues were easily peeled)	1
	Serious adhesion	2
Inflammation and ulceration	No ulceration and inflammation	0
	Local congestion and no ulceration	1
	1 ulcer without congestion or thickening of intestinal wall	2
	1 ulcer with inflammation	3
	≥ 2 ulcers with inflammation	4
	> 2 ulcers and > 1 cm inflamed area	5
	Ulcers with > 2 cm inflamed area and increasing 1 score for each additional 1 cm lesion	6–8

### 2.9 In Vivo Imaging

Mice were anaesthetised with 1.5% isoflurane. The anaesthetised mice were intraperitoneally injected with 150 mg/kg D‐Luciferin Potassium Salt (PerkinElmer, USA). After 12 min of injection, the total signal produced by Luc‐labelled transplanted cells was imaged under IVIS Spectrum in vivo imaging system (PerkinElmer).

### 2.10 Immunohistochemistry (IHC)

Following dewaxing and hydration, paraffin sections were subjected to antigen retrieval. Sections were blocked with hydrogen peroxide blocking solution. After that, the sections were incubated with α‐smooth muscle actin (α‐SMA) (1:10,000 dilution; Cat#67735‐1‐Ig; Proteintech, Wuhan, China) at 4°C overnight, and then treated with HRP‐IgG (1:2000 dilution; Cat#ab205719; Abcam; Cambridge, MA, USA) at 37°C for 1 h. The sections were stained with DAB and counterstained with haematoxylin. The images were observed under a confocal laser scanning microscope (Nikon, Tokyo, Japan).

### 2.11 Flow Cytometry

Colon tissues were digested with Collagenase I (Sigma‐Aldrich, St. Louis, MO, USA) to prepare cell suspension. Cells were fixed with IC Fixation Buffer (Thermo Fisher Scientific) for 30 min, and permeabilized with 1× Permeabilization Buffer (Thermo Fisher Scientific) for 30 min. The cell suspension was incubated with FITC anti‐mouse CD4 antibody (Cat#100405; BioLegend, San Diego, CA, USA), APC anti‐mouse CD194 antibody (CCR4) (Cat#131203; BioLegend), Alexa Fluor 647 anti‐mouse FOXP3 antibody (Cat#126407; BioLegend), CoraLite Plus 647 anti‐α‐SMA antibody (Cat#CL647‐67735; Proteintech, Wuhan, China) and FITC anti‐mouse F4/80 antibody (Cat#123107; BioLegend) in the dark for 1 h at 4°C. Cells were incubated with isotype‐matched antibodies and unstained cells were used as negative controls. After that, the single cells were analysed by FACSCalibur flow cytometer (BD Biosciences, San Jose, CA, USA).

### 2.12 Histochemical Analysis

Paraffin sections (4 μm) of intestinal tissue and colon tissue samples were subjected to dewaxing and hydration. Haematoxylin and eosin (H&E) Stain Kit and Masson's Trichrome Stain Kit were purchased from Solarbio (Beijing, China). According to the manufacturer, these kits were used to evaluate the pathological changes and fibrosis of intestinal tissue and colon tissue. The sections were observed under an optical microscope. The fibrosis area of intestinal tissue was calculated by ImageJ software (National Institutes of Health, Bethesda, MD, USA).

### 2.13 Co‐Immunoprecipitation (Co‐IP) Assays

Cells were treated with RIPA Lysis and Extraction Buffer (Thermo Fisher Scientific). Cell lysate was incubated with anti‐CCL17 (1:1000 dilution; Cat#DF9913; Affinity) at 4°C and 20 rpm overnight. After that, Protein A/G agarose beads (Thermo Fisher Scientific) were added to the cell lysate, incubated at 4°C and 20 rpm for 4 h. The proteins were eluted from Protein A/G agarose bead‐protein complex, and then denatured at 100°C for 10 min. The eluted proteins were analysed by WB.

### 2.14 Enzyme‐Linked Immunosorbent Assay (ELISA)

The level of CCL17 in cell supernatant and colon tissues was examined by ELISA using Mouse CCL17/TARC ELISA Kit (Weiaobio, Shanghai, China). The samples were seeded into enzyme plates and cultured at 37°C for 1 h, and then incubated with the chromogenic antibody for 30 min. Finally, the absorbance value at 450 nm was measured by a microplate reader (Thermo Fisher Scientific, San Jose, CA, USA).

### 2.15 WB

Total proteins were extracted from cells and tissues utilising RIPA reagent (Thermo Fisher Scientific). Total proteins were extracted from sEVs by applying Total Exosome RNA and Protein Isolation Kit (Thermo Fisher Scientific). BCA Protein Assay Kit (Biosharp, Hefei, China) was used to detect the concentration of proteins. Then, 10% SDS‐PAGE protein electrophoresis was carried out to separate protein samples. The separated proteins were transferred onto polyvinylidene difluoride membranes. The membranes were incubated with anti‐α‐SMA (1:20,000 dilution; Cat#67735‐1‐Ig; Proteintech), anti‐collagen type I alpha 1 (COL1A1; 1:1000 dilution; Cat#ab270993; Abcam), anti‐TGF‐β1 (1:1000 dilution; Cat#ab215715; Abcam), anti‐p38 MAPK (1:1000 dilution; Cat#ab308333; Abcam), anti‐p‐p38 MAPK (1:1000 dilution; Cat#28796‐1‐AP; Proteintech), anti‐CCL17 (1:1000 dilution; Cat#ab182793; Abcam), anti‐CCR4 (1:1000 dilution; Cat#ab216560; Abcam), anti‐CD63 (1:1000 dilution; Cat#25682‐1‐AP; Proteintech), anti‐MFGE8 (1:1000 dilution; Cat#PA5‐109955; Thermo Fisher Scientific), anti‐Fibronectin 1 (FN1; 1:2000; Cat#15613‐1‐AP; Proteintech), anti‐Vimentin (VIM; 1:20000; Cat#A19607; ABclonal) or anti‐GAPDH (1:5000 dilution; Cat#10494‐1‐AP; Proteintech) at 4°C overnight. The membranes were stained with HRP‐IgG (1:2000 dilution; Cat#ab205718; Abcam) at 37°C for 1 h. The immunoreactive bands were analysed by ImageJ software.

### 2.16 Immunofluorescence (IF) Staining

Cells were cultured on glass‐bottom dishes and then fixed with 4% paraformaldehyde. The fixed cells were subjected to antigen retrieval and goat serum blocking. The fixed cells were incubated with rat anti‐mouse CD68 (1:50 dilution; Cat#ab53444; Abcam), rabbit anti‐mouse α‐SMA (1:500 dilution; Cat#ab124964; Abcam) or rabbit anti‐mouse CCR4 (1:100 dilution; Cat#PA1‐41155; Thermo Fisher Scientific) at 4°C overnight. For intracellular protein staining, the fixed cells were treated with Permeabilization Wash Buffer (Yeasen, Shanghai, China). After that, cells were stained with rat anti‐mouse FoxP3 (1:100 dilution; Cat#41‐5773‐82; Thermo Fisher Scientific) at 4°C overnight. Cells were stained with secondary antibodies goat anti‐rat IgG‐Alexa Fluor 647 (1:500 dilution; Cat#ab150167; Abcam), goat anti‐rabbit IgG‐Alexa Fluor 488 (1:500 dilution; Cat#ab150077; Abcam), donkey anti‐rabbit IgG‐Alexa Fluor 647 (1:500 dilution; Cat#ab150075; Abcam) or goat anti‐rat IgG‐Alexa Fluor 488 (1:500 dilution; Cat#ab150165; Abcam) at 37°C for 1 h. Nuclei were stained with DAPI. The α‐SMA^+^CD68^+^ positive cells and FoxP3^+^CCR4^+^ positive cells were observed through confocal laser scanning microscopy.

### 2.17 Quantitative Real‐Time PCR (qRT‐PCR)

Total RNA was extracted from cells and sEVs using TRIzol reagent (Thermo Fisher Scientific) or Total Exosome RNA and Protein Isolation Kit (Thermo Fisher Scientific). cDNA was synthesised using PrimeScript RT reagent Kit with gDNA Eraser (Perfect Real Time) (Takara, Beijing, China). Gene expression was detected by qPCR using SYBR Premix EX Taq kit (Takara). The expression levels of mRNAs were normalised to GAPDH. The relative expression levels of the genes were calculated using the 2^−ΔΔCT^ method. The primer sequences (5′–3′) used in the present study are as follows: CCL17, forward‐CGA GAG TGC TGC CTG GAT TAC T and reverse‐GGT CTG CAC AGA TGA GCT TGC C; CCR4, forward‐GGA CTA GGT CTG TGC AAG ATC G and reverse‐TGC CTT CAA GGA GAA TAC CGC G; MFGE8, forward‐CGG GCC AAG ACA ATG ACA TC and reverse‐TCT CTC AGT CTC ATT GCA CAC AAG; GAPDH, forward‐AAT GGA CAA CTG GTC GTG GAC and reverse‐CCC TCC AGG GGA TCT GTT TG.

### 2.18 Co‐Culture of Tregs and Mø

First, Tregs were isolated from spleen of mice and ground through a 70‐μm filter to obtain a single‐cell suspension. Then, Dynabeads Mouse T‐Activator CD3/CD28 (Thermo Fisher Scientific) were incubated with Tregs at a 1:1 ratio, and 100 U/mL IL‐2 (Thermo Fisher Scientific) was added to maintain the culture for Treg selection and activation. Finally, Transwell chamber (Corning, NY, USA) was used for co‐culture. Tregs were seeded into the lower chamber containing DMEM and 10% FBS; Mø were seeded into the upper chamber.

### 2.19 Cell Proliferation and Migration

Cell viability and wound‐healing assays detected proliferation and migration of Tregs. For cell proliferation, Tregs (2000 cells/100 μL) were incubated with 10 μL Cell Counting Kit‐8 (CCK‐8) reagent (Beyotime, Shanghai, China) at 37°C for 1 h. The absorbance value of cells at 450 nm was measured by a microplate reader (Thermo Fisher Scientific). For cell migration, Tregs (6 × 10^5^) were plated into 6‐well plates and incubated at 37°C. When cells reached > 90% confluence, a vertical scratch was made by a 10 μL tip. Cells were further cultured in an FBS‐free medium. The width of the scratch was observed at 0 and 24 h using an inverted light microscope.

### 2.20 Statistical Analysis

Each assay was performed three times. Data were analysed by SPSS 22.0 statistical software (IBM, Armonk, NY, USA) and expressed as mean ± standard deviation. The two groups' differences were analysed using an unpaired two‐tailed Student's *t*‐test. If there were more than two groups, one‐way or two‐way ANOVA with Tukey's post hoc analysis was used. *p* < 0.05 was considered a significant difference.

## 3 Results

### 3.1 MMT and Tregs Infiltration Were Observed in CD Patients

**FIGURE 1:**
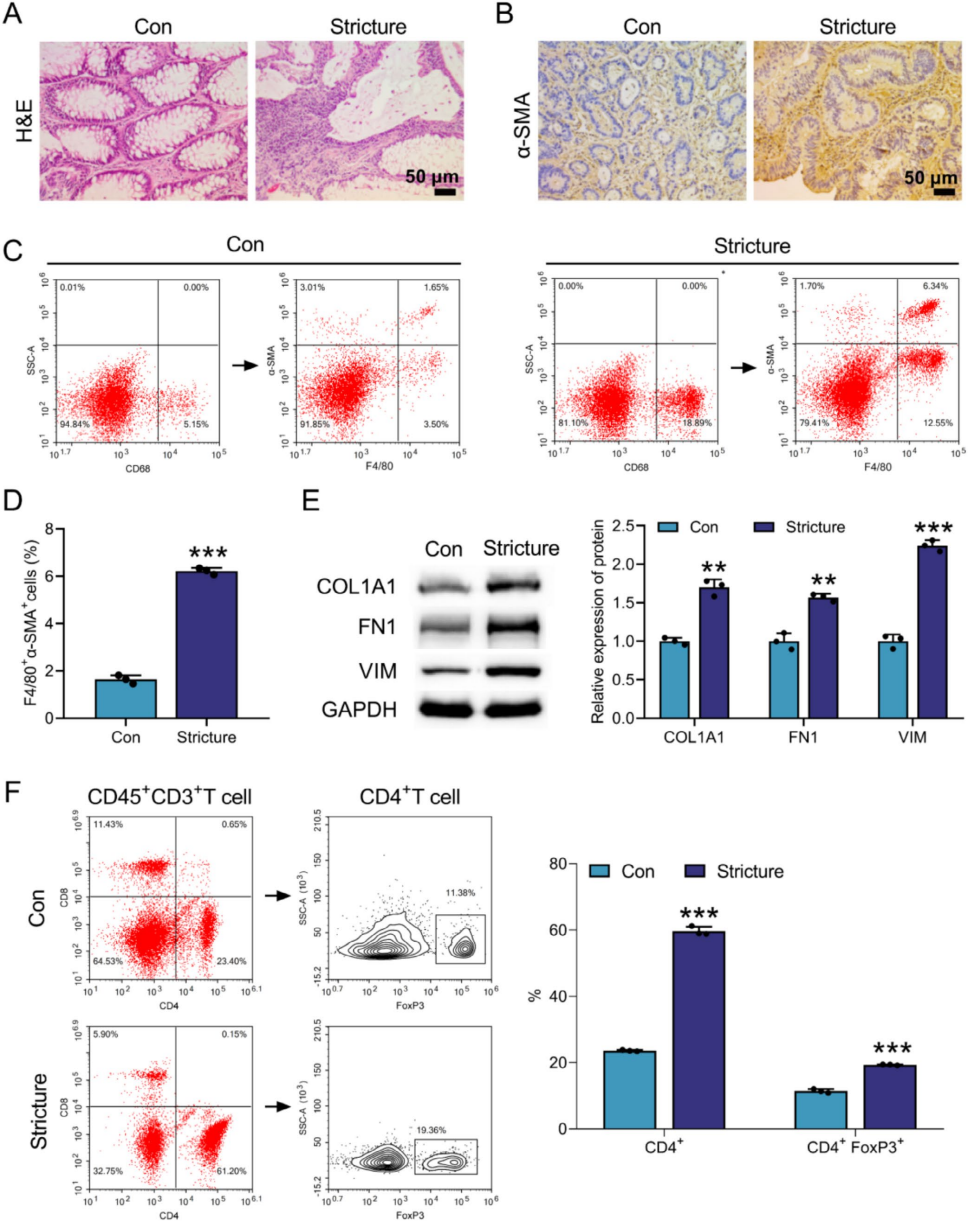
Infiltration of Mø‐derived myofibroblasts and Tregs was observed in CD patients. Stricturing intestinal tissue and adjacent normal intestinal tissue samples were obtained from CD patients. Experimental groups: Con and Stricture. (A) H&E staining was used to evaluate the pathological changes of intestinal tissues. (B) IHC staining was performed to examine the expression of α‐SMA in intestinal tissues. (C and D) Flow cytometry was employed to assess the proportions of F4/80^+^α‐SMA^+^ Mø in intestinal tissues. (E) The protein levels of COL1A1, FN1 and VIM were measured by WB. (F) The proportions of CD4^+^ and FoxP3^+^ Treg cells in intestinal tissues were tested by flow cytometry. Data are presented as mean ± SD, *n* = 3. **p < 0.01 and ***p < 0.001 versus Con group.

To demonstrate whether there was MMT and Treg cell infiltration in CD patients, we first evaluated the pathological changes in stricturing intestinal tissues of CD patients by H&E staining. In non‐stenotic intestinal tissues, the intestinal mucosa was intact, with well‐arranged glands and no inflammatory cell infiltration. In contrast, the stricturing intestinal tissues of CD patients showed deformation and distortion of the intestinal mucosal glands, as well as missing glands and a large infiltration of inflammatory cells (Figure 1A). Next, the expression of myofibroblast marker α‐SMA in intestinal tissues was examined. IHC assay revealed that α‐SMA expression was significantly increased in stricturing intestinal tissues compared with non‐stenotic intestinal tissues (Figure 1B). Flow cytometry further detected a significant increase in the proportion of F4/80^+^α‐SMA^+^ Mø in the Stricture group, indicating enhanced MMT (Figure 1C,D). WB analysis showed that the protein expression levels of COL1A1, FN1 and VIM were significantly upregulated in the Stricture group compared to the Con group (Figure 1E). Interestingly, flow cytometry results showed that the proportions of CD4^+^ cells and CD4^+^FoxP3^+^ cells (Tregs) were elevated in stricturing intestinal tissues of CD patients (Figure 1F). These data indicated that CD patients exhibited increased infiltration of Mø‐derived myofibroblasts and Tregs.

### 3.2 CD Mice Exhibited MMT and Increased Infiltration of Tregs

**FIGURE 2:**
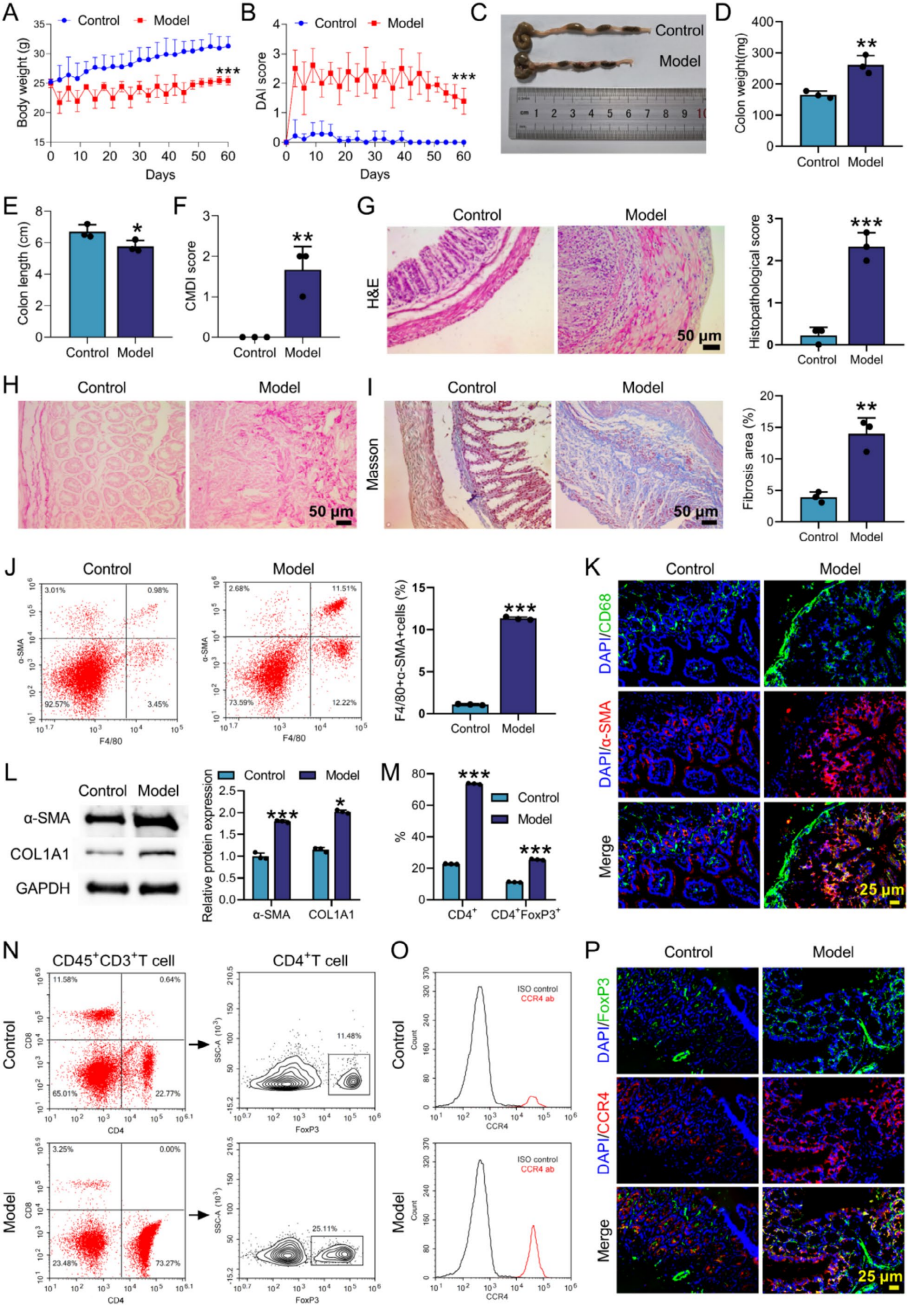
Increased infiltration of Mø‐derived myofibroblasts and Tregs was observed in CD mice. CD mouse model was constructed by TNBS. Normal mice served as controls. (A) Body weight changes. (B) DAI score. (C) Images of colon tissues. (D and E) Weight and length of colon tissues. (F) CMDI score. (G) H&E staining was performed to evaluate the pathological changes of colon tissues. (H and I) Sirius Red and Masson staining were conducted to measure the extent of colon tissue fibrosis. (J) Flow cytometry was used to assess the proportions of F4/80^+^α‐SMA^+^ Mø in colon tissues. (K) IF staining with CD68 and α‐SMA was utilised to assess the distribution of Mø‐derived myofibroblasts. (L) WB was performed to examine the expression of α‐SMA and COL1A1 in colon tissues. (M–O) The proportion of CD4^+^, FoxP3^+^Treg cells and CCR4 expression in colon tissues was evaluated using flow cytometry. (P) IF staining with CCR4 and FoxP3 was applied to evaluate the expression of CCR4 in Treg of colon tissues. Data are presented as mean ± SD, *n* = 3. *p < 0.05, **p < 0.01 and ***p < 0.001 versus Control group.

To test the MMT in vivo, CD mouse model was established by TNBS administration. Compared with the control group, CD mice exhibited body weight loss, increased DAI score, elevated colon weight, shorter colon length and significantly higher CMDI score (Figure 2A–F). The pathological changes in colon tissues were evaluated by H&E staining. The mice in the Control group exhibited intact intestinal mucosa, whereas CD mice showed disrupted mucosal integrity, with a large number of inflammatory cells infiltrating the mucosal layer. Additionally, some muscle layers exhibited thickening whilst inflammatory cells infiltrated (Figure 2G). Both Sirius red and Masson staining showed significantly greater collagen deposition in the model group, indicating increased tissue fibrosis (Figure 2H,I). Flow cytometry analysis revealed that the proportion of F4/80^+^α‐SMA^+^ Mø in the model group was significantly higher than that in the control group (Figure 2J). IF assay confirmed that the fluorescence intensity of α‐SMA^+^CD68^+^ cells and the expression level of CD68 were significantly higher in the model group than in the control group, and the fluorescence expression of CD68 was significantly higher than that of α‐SMA, suggesting that the conversion of Mø to myofibroblasts was enhanced under pathological conditions, but the converted cells still retained strong Mø characteristics (Figure 2K). Furthermore, WB analysis confirmed significant up‐regulation of myofibroblast markers α‐SMA and COL1A1 in the colonic tissues of CD mice compared to controls (Figure 2L). Meanwhile, flow cytometry and IF double‐labelling revealed that the proportion of FoxP3^+^ Treg cells and the expression of CCR4 on their surface were significantly increased in CD mice, with the fluorescence intensity of FoxP3 significantly higher than that of CCR4 (Figure 2M–P). These findings demonstrated that increased infiltration of Mø‐derived myofibroblasts and Tregs was observed in CD mice.

### 3.3 Mø Inoculation Elevated the MMT and the Proportions of Tregs in CD Mice

**FIGURE 3:**
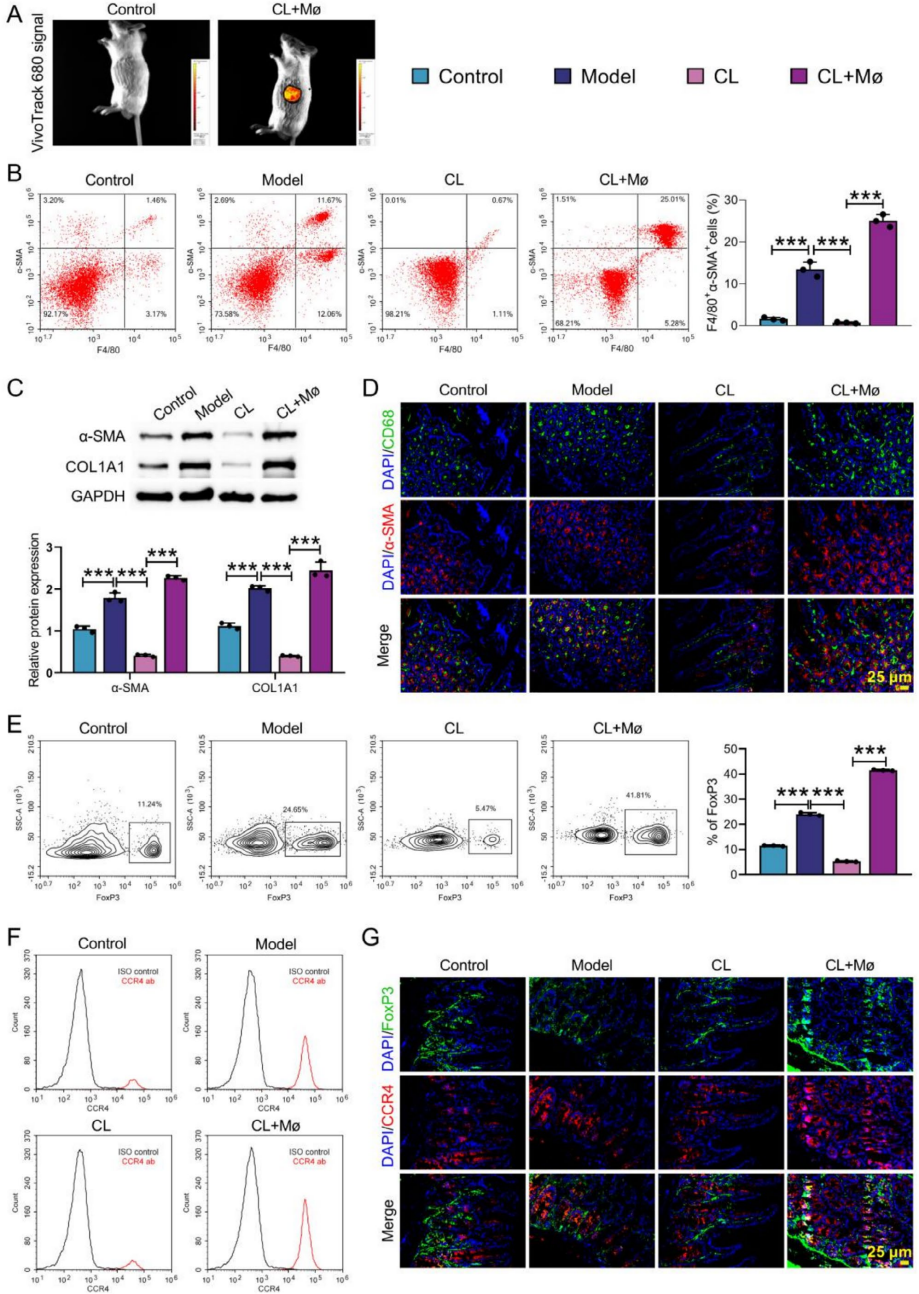
Mø inoculation enhanced the MMT and the proportions of Tregs in CD mice. CD mouse model was constructed by TNBS, and then administered with CL and CL + Mø. (A) In vivo imaging of mice was utilised to observe the distribution of exogenous Mø in the colon. (B) Flow cytometry was conducted to assess the proportions of F4/80^+^α‐SMA^+^ Mø in colon tissues. (C) WB was used to test the protein expression levels of α‐SMA and COL1A1. (D) IF staining with CD68 and α‐SMA was applied to evaluate the distribution of Mø‐derived myofibroblasts. (E and F) Flow cytometry was utilised to detect the expression of CCR4 and the proportion of FoxP3^+^Treg cells in colon tissues. (G) IF staining with CCR4 and FoxP3 was conducted to evaluate the expression of CCR4 in FoxP3^+^Treg cells of colon tissues. Data are presented as mean ± SD, *n* = 3. ***p < 0.001 versus Control or Model or CL.

To investigate MMT in vivo, CD mice were injected with Mø. In vivo imaging revealed that the exogenous Mø localised to the colon tissues of CD mice (Figure 3A). Flow cytometry assay revealed that the proportion of F4/80^+^α‐SMA^+^ Mø was significantly higher in the Model groups, Mø scavenger CL treatment notably reduced the proportions of F4/80^+^α‐SMA^+^ cells, whilst Mø inoculation caused a significant increase in proportions of F4/80^+^α‐SMA^+^ cells in CD mice (Figure 3B). WB analysis confirmed that, compared with the control group, the protein levels of myofibroblast markers α‐SMA and COL1A1 were significantly elevated in the model group, whilst Mø inoculation reversed CL treatment‐mediated inhibition of these protein levels (Figure 3C). IF double staining showed that the proportion of α‐SMA^+^CD68^+^ double‐positive cells was significantly higher in the model group than in the control and CL groups, and significantly increased in the CL + Mø group compared to the CL group. Notably, the fluorescence intensity of CD68 was higher than that of α‐SMA, indicating that some Mø had not yet fully differentiated into myofibroblasts (Figure 3D). Further analysis of Treg cell subpopulations demonstrated that the proportion of FoxP3^+^ Treg cells and their surface CCR4 expression were significantly upregulated in the Model and CL + Mø groups. Notably, the proportion of FoxP3^+^CCR4^+^ double‐positive cells in the CL + Mø group was slightly higher than that in the Model group, whereas almost no double‐positive cells were detected in the Con and CL groups (Figure 3E,F). Meanwhile, IF double‐labelling further confirmed these results (Figure 3G). These findings revealed that Mø inoculation enhanced the MMT and the proportions of Tregs in CD mice.

### 3.4 TGF‐β1 Treatment Induced MMT by Activating MAPK Signalling Pathway

**FIGURE 4:**
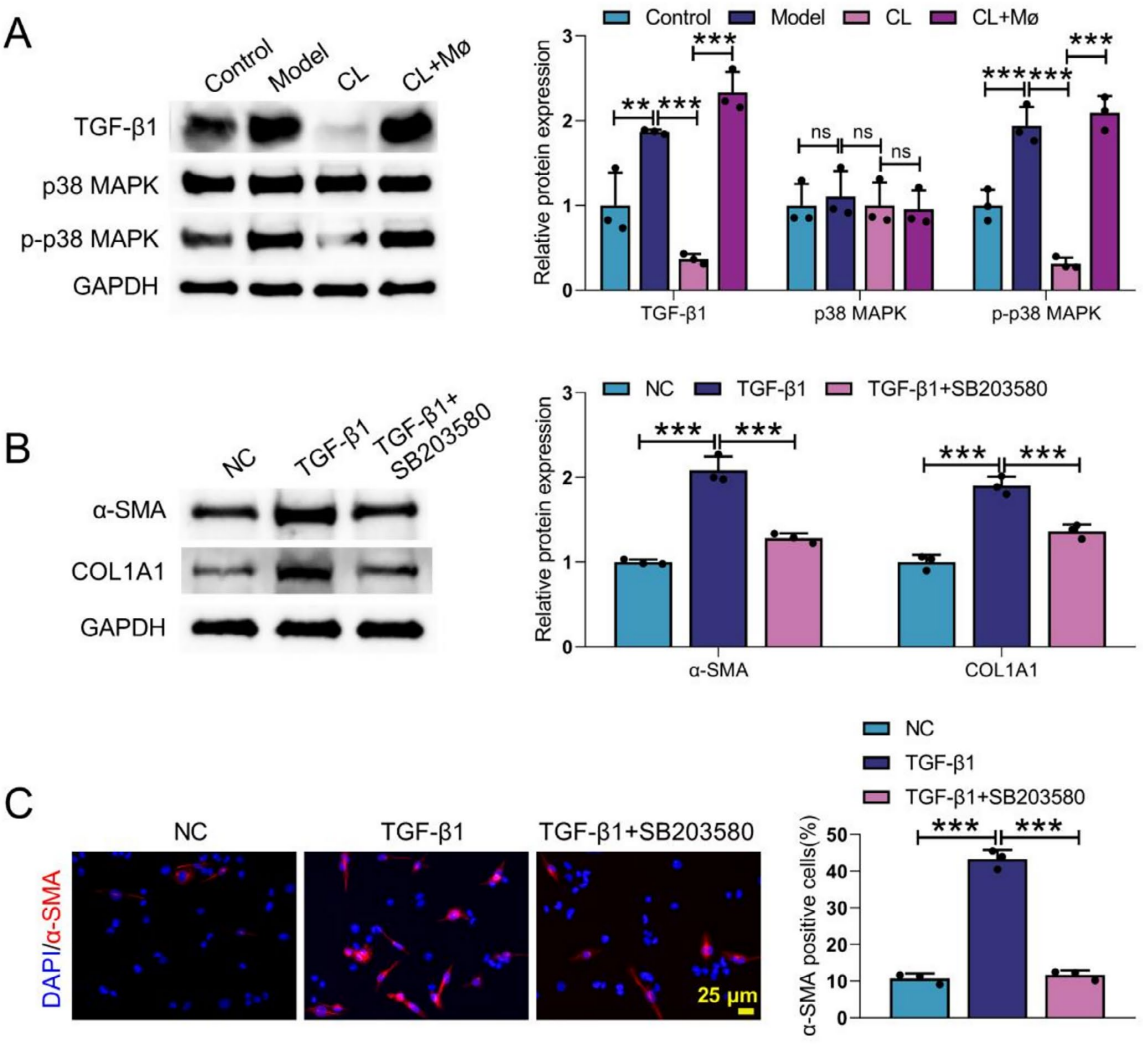
SB203580 reversed TGF‐β1‐mediated MMT. CD mouse model was constructed by TNBS, and then administered with CL and CL + Mø. In vivo experimental groups: Control, Model, CL and CL + Mø. (A) The expression levels of TGF‐β1, p38 MAPK and p‐p38 MAPK in colon tissues were detected by WB. Mø were treated with TGF‐β1 or TGF‐β1 + SB203580. (B) WB was employed to detect the protein expression of α‐SMA and COL1A1 in Mø. (C) IF assay was utilised to assess the expression of α‐SMA in Mø. Data are presented as mean ± SD, *n* = 3. **p < 0.01 and ***p < 0.001 versus Control or Model or CL or NC or TGF‐β1. ns means no significant difference.

MAPK signalling pathway mediates TGF‐β‐induced collagen expression and fibrosis occurrence [23]. Here, we examined the activity of the MAPK signalling pathway in CD mice by WB. The expression levels of TGF‐β1 and p‐p38 MAPK were elevated in CD mice, whilst CL treatment downregulated their expression levels. Notably, Mø treatment significantly reversed the CL treatment‐mediated suppression (Figure 4A). In cellular experiments, TGF‐β1 was used to induce MMT in Mø. TGF‐β1 treatment elevated the expression levels of α‐SMA and COL1A1 in Mø, which were inhibited by the MAPK inhibitor SB203580 (Figure 4B). IF labelling further showed that the TGF‐β1 + SB203580 group significantly reduced the TGF‐β1‐induced increase in the proportion of α‐SMA^+^ cells, further supporting the above conclusion (Figure 4C). Thus, these results indicated that TGF‐β1 treatment induced MMT by activating the MAPK signalling pathway.

### 3.5 TGF‐β1‐Induced MMT Recruited Tregs Through the Interaction Between CCL17 and CCR4

**FIGURE 5:**
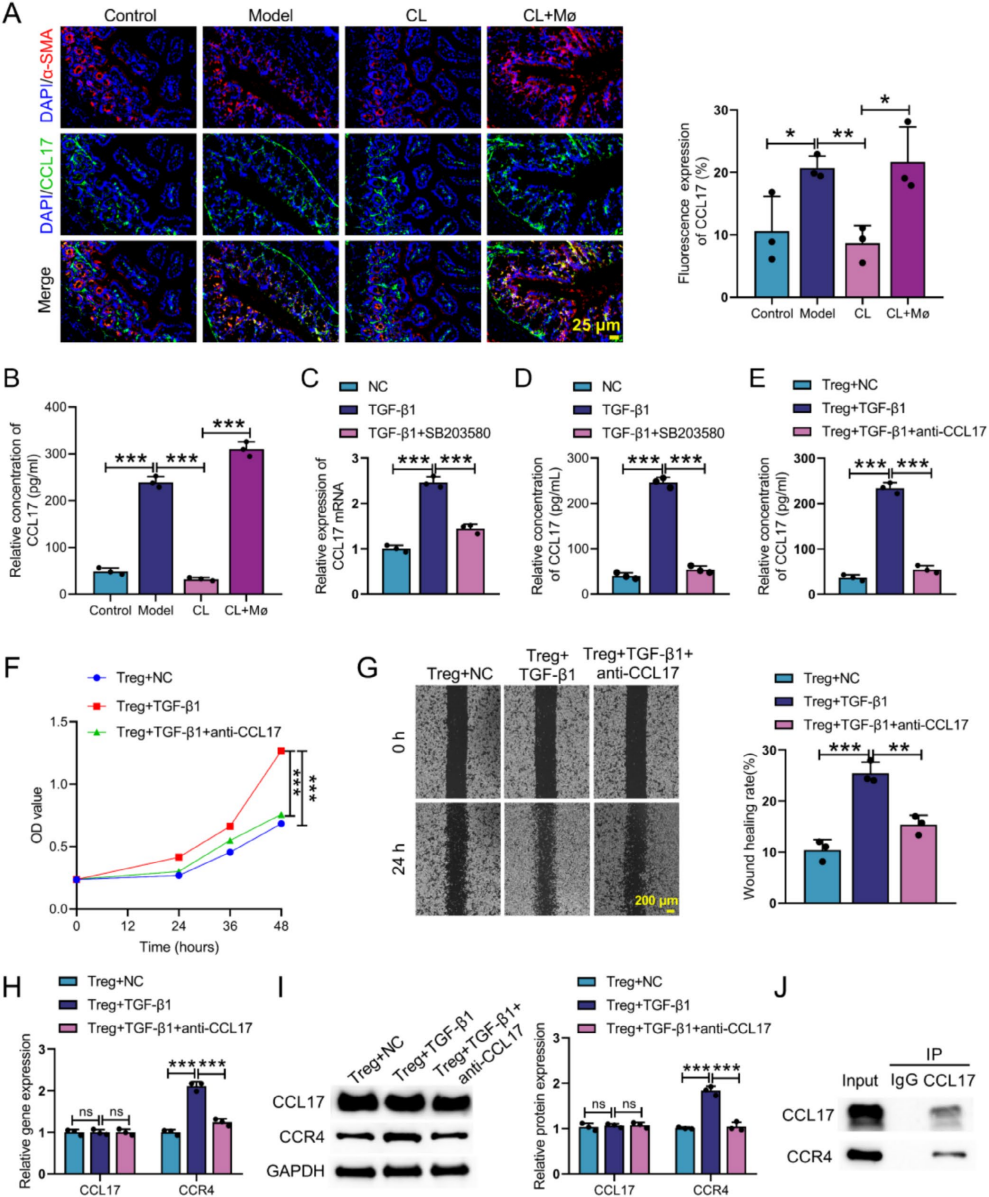
TGF‐β1‐induced transdifferentiation of myofibroblasts recruited Tregs through the interaction between CCL17 and CCR4. CD mouse model was constructed by TNBS, and then administered with CL and CL + Mø. In vivo experimental groups: Control, Model, CL and CL + Mø. (A) IF staining was utilised to assess the expression of CCL17 and α‐SMA in colon tissues. (B) The level of CCL17 in colon tissues was detected by ELISA. Isolation of spleen mononuclear Mø from healthy mice; in vitro experiments were divided into: NC, TGF‐β1 and TGF‐β1 + SB203580. (C and D) qRT‐PCR and ELISA were performed to examine the expression level of CCL17. Mø and Treg cells were co‐cultured; the experimental groups were divided into: Treg + NC, Treg + TGF‐β1 and Treg + TGF‐β1 + anti‐CCL17. (E) The CCL17 level was tested in Treg supernatant by ELISA. (F and G) CCK‐8 and wound‐healing assays were used to detect cell proliferation and migration. (H and I) qRT‐PCR and WB were conducted to examine the expression of CCL17 and CCR4. (J) Co‐IP was conducted to verify the interaction between CCL17 and CCR4. Data are presented as mean ± SD, *n* = 3. *p < 0.05, **p < 0.01 and ***p < 0.001 versus Control or Model or CL or NC or TGF‐β1 or Treg + NC or Treg + TGF‐β1. ns means no significant difference.

To further investigate whether MMT‐derived myofibroblasts recruit Treg cells via CCL17 secretion, we conducted both in vitro and in vivo experiments. In vivo experiments, IF analysis showed a significant increase in CCL17 and α‐SMA fluorescence intensity in the model compared to the control and CL groups, and Mø inoculation also caused a significant increase. Quantitative assessment showed that the CL + Mø group had the strongest CCL17 signal, followed by the model group, whilst the control and CL groups had less CCL17 fluorescence. Notably, α‐SMA fluorescence intensity was significantly higher than CCL17 fluorescence intensity, and the fluorescence trend of α‐SMA was consistent with that of CCL17 (Figure 5A). ELISA results showed that CL treatment inhibited the level of CCL17 in CD mice, whilst CL + Mø treatment restored CCL17 production (Figure 5B). In the TGF‐β1‐induced Mø, CCL17 mRNA level and supernatant CCL17 content were significantly higher in the TGF‐β1‐intervened group than in the NC group, whereas this effect was inhibited by the addition of SB203580 (Figure 5C,D). Then, Mø were co‐cultured with Tregs. ELISA results showed that the addition of CCL17‐neutralising antibody (anti‐CCL17) led to a significant decrease of CCL17 in Treg cell supernatant compared with the TGF‐β1‐treated Mø and Treg cell co‐culture group (Figure 5E). CCK‐8 and wound‐healing assays demonstrated that TGF‐β1‐treated Mø promoted proliferation and migration of Treg, which was reversed by anti‐CCL17 (Figure 5F,G). qRT‐PCR and WB results revealed that TGF‐β1‐treated Mø caused an up‐regulation of CCR4 in Tregs, whilst anti‐CCL17 treatment decreased the CCR4 expression level (Figure 5H,I). Moreover Co‐IP assay demonstrated that CCL17 interacted with CCR4 (Figure 5J). All these findings demonstrated that TGF‐β1‐induced transdifferentiation of myofibroblasts recruited Tregs through the interaction between CCL17 and CCR4.

### 3.6 AMSC‐sEVs Inhibited MMT by Delivering MFGE8

**FIGURE 6:**
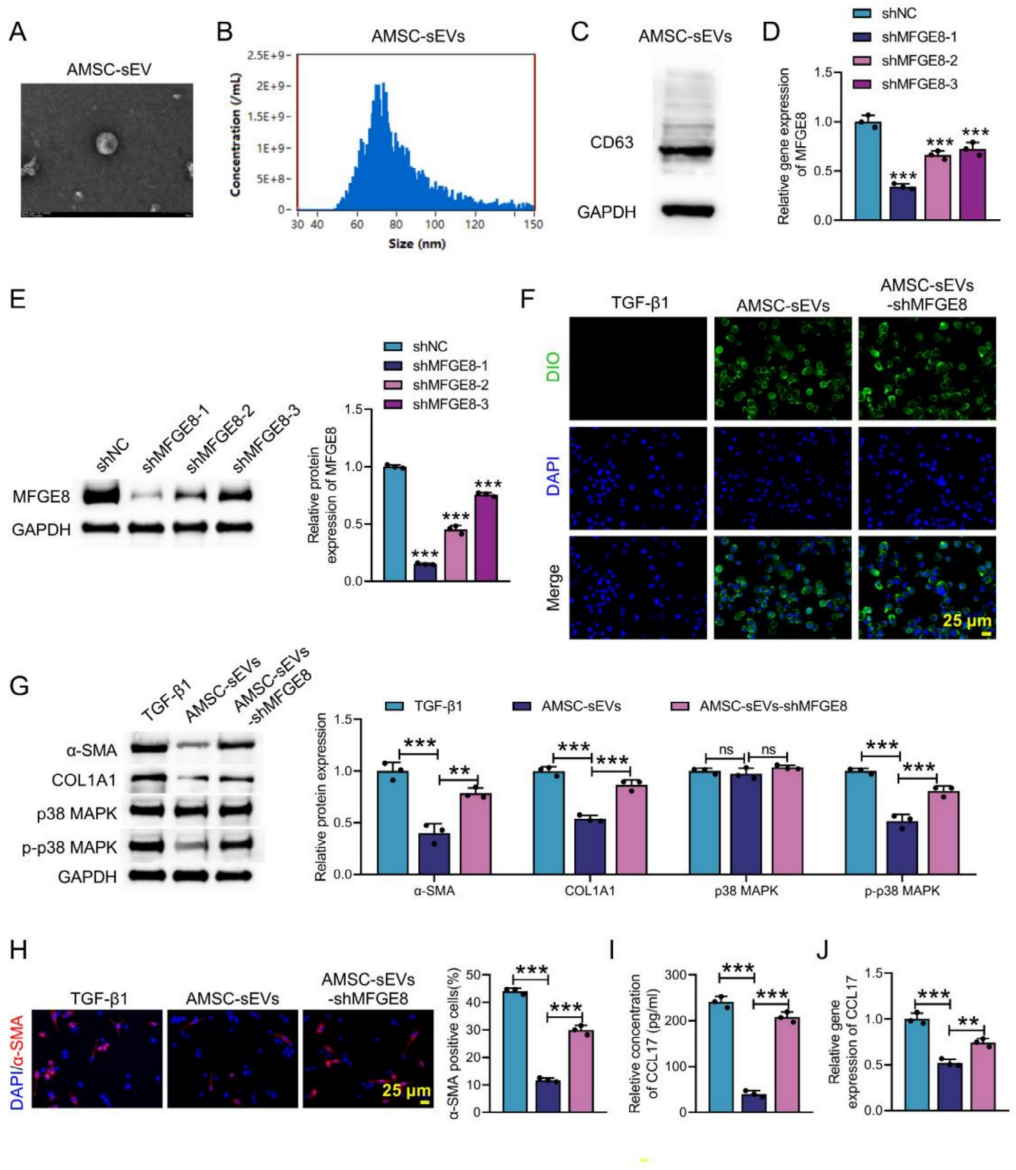
AMSC‐sEVs inhibited MMT by delivering MFGE8. AMSCs were cultured in vitro. (A) TEM was employed to observe the exosome characteristics of AMSC‐sEVs. (B) NTA assay was utilised to detect the concentration and diameter of particles. (C) WB was used to assess the expression of CD63 in AMSC‐sEVs. AMSCs were treated with shMFGE8‐1, shMFGE8‐2 or shMFGE8‐3. (D and E) qRT‐PCR and WB were used to examine the expression of MFGE8 in AMSC‐sEVs. AMSC‐sEVs were co‐cultured with Mø, and the experimental groups were divided into: TGF‐β1, AMSC‐sEVs and AMSC‐sEVs‐shMFGE8. (F) Exosomes entering Mø were observed by DIO dye. (G) WB was utilised to detect the expression of α‐SMA, COL1A1, p38 MAPK and p‐p38 MAPK. (H) IF staining with α‐SMA was conducted to assess the proportion of α‐SMA‐positive cells. (I and J) The level of CCL17 was evaluated using ELISA and qRT‐PCR. Data are presented as mean ± SD, *n* = 3. **p < 0.01 and ***p < 0.001 versus sh‐NC or TGF‐β1 or AMSC‐sEVs. ns means no significant difference.

Our previous research has confirmed that AMSC‐sEVs improve intestinal fibrosis in CD mice by delivering MFGE8. The AMSC‐sEVs displayed a disc‐shaped membrane structure with a diameter ranging from 50 to 150 nm, as determined by TEM and NTA analysis (Figure 6A,B). WB analysis confirmed the sEV marker CD63 was abundant in AMSC‐sEVs (Figure 6C). To further explore the functional role of MFGE8, AMSC‐sEVs were treated with shMFGE8‐1, shMFGE8‐2 or shMFGE8‐3. qRT‐PCR and WB analysis confirmed that shMFGE8‐1 exhibited the highest knockdown efficiency in AMSC‐sEVs amongst the three sequences, so we chose shMFGE8‐1 for further study (Figure 6D,E). Then, Mø were incubated with AMSC‐sEVs. DIO dye labelling showed that AMSC‐sEVs could successfully enter Mø (Figure 6F). WB analyses showed that AMSC‐sEVs significantly inhibited TGF‐β1‐induced α‐SMA, COL1A1 and p‐p38 MAPK expression, which was partially reversed by MFGE8 knockdown (Figure 6G). IF analysis further revealed that the proportion of α‐SMA^+^ cells was reduced in the AMSC‐sEVs; shMFGE8 treatment led to an increase in the proportion of α‐SMA^+^ cells in Mø (Figure 6H). Furthermore, ELISA and qRT‐PCR assays showed that AMSC‐sEVs significantly reduced the supernatant CCL17 content and CCL17 mRNA level, whilst MFGE8 knockdown attenuated this effect (Figure 6I,J). Taken together, these findings showed that AMSC‐sEVs inhibited MMT by delivering MFGE8.

### 3.7 AMSC‐sEVs Delivered MFGE8 to Inhibit MMT and Intestinal Fibrosis in CD Mice

**FIGURE 7:**
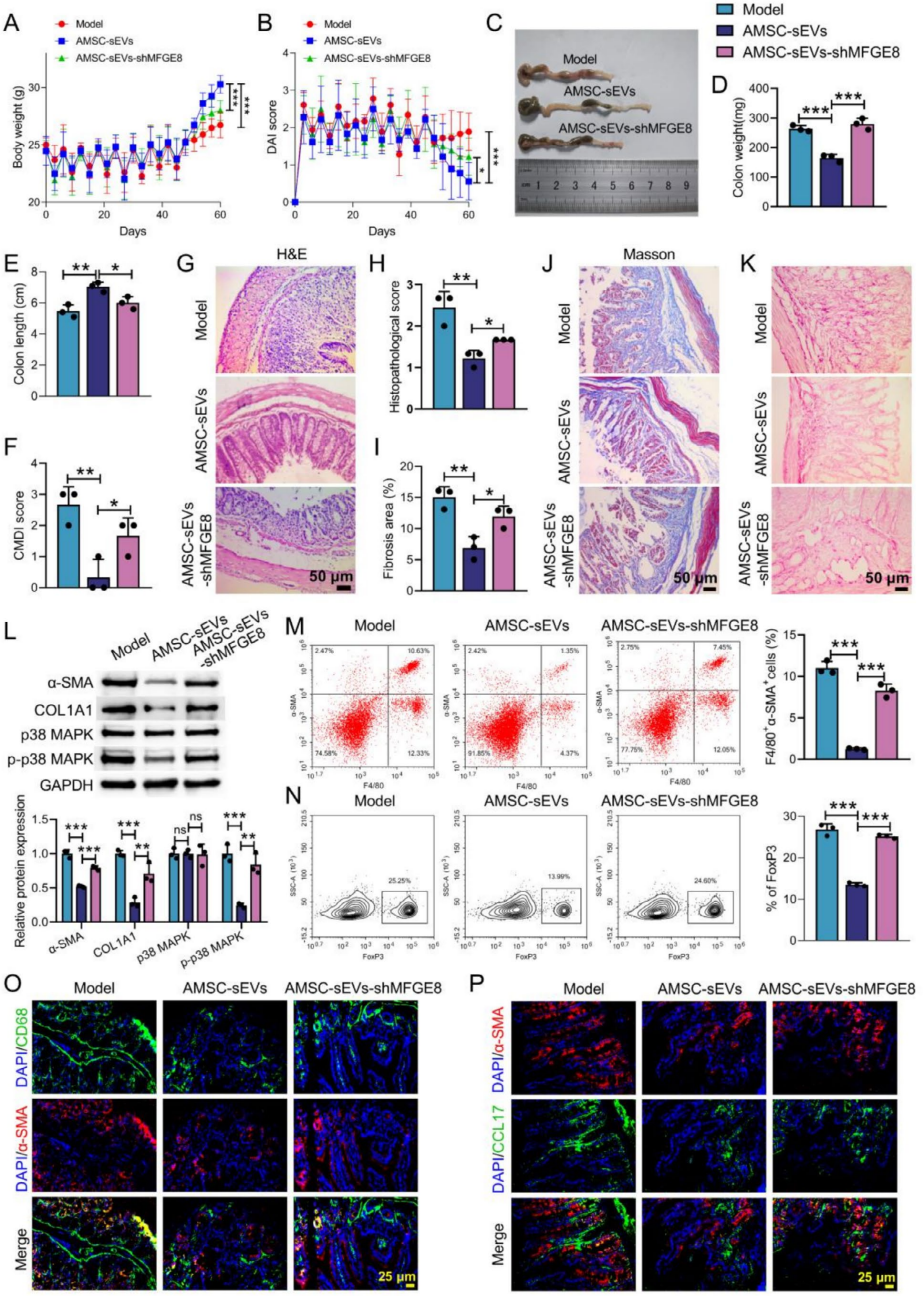
AMSC‐sEVs delivered MFGE8 to inhibit MMT and intestinal fibrosis in CD mice. CD mouse model was constructed by TNBS, and then administered with AMSC‐sEVs and AMSC‐sEVs‐shMFGE8. (A) Body weight changes. (B) DAI score. (C) Images of colon tissues. (D and E) Weight and length of colon tissues. (F) CMDI score. (G and H) H&E staining was used to evaluate the pathological changes of colon tissues. (I–K) Masson and Sirius Red staining were conducted to measure the extent of tissue fibrosis. (L) The α‐SMA, COL1A1, p38 MAPK and p‐p38 MAPK protein levels were tested by WB. (M and N) Flow cytometry was employed to assess the proportions of F4/80^+^α‐SMA^+^ Mø and FoxP3^+^Treg in colon tissues. (O) IF staining with CD68 and α‐SMA was performed to assess the distribution of Mø‐derived myofibroblasts. (P) IF staining with α‐SMA and CCL17 was used to evaluate the expression of CCL17 in myofibroblasts of colon tissues. Data are presented as mean ± SD, *n* = 3. * *p* < 0.05, **p < 0.01 and ***p < 0.001 versus Model or AMSC‐sEVs. ns means no significant difference.

Finally, we investigated the functional role of AMSC‐sEVs in CD mice in vivo. AMSC‐sEVs significantly improved body weight, DAI score, colon weight, colon length and CMDI score, whereas AMSC‐sEVs‐shMFGE8 exhibited the opposite impact on CD mice (Figure 7A–F). Moreover, H&E staining results showed that the integrity of the intestinal mucosa in CD mice was disrupted, with a large number of inflammatory cells infiltrating the mucosal layer. Some muscle layers exhibited thickening whilst inflammatory cells infiltrated. There was less disruption of intestinal mucosal integrity and less inflammatory cell infiltration in the AMSC‐sEVs group. Compared with the AMSC‐sEVs group, AMSC‐sEVs‐shMFGE8 caused severe damage to colon tissues in CD mice (Figure 7G,H). Results obtained from Masson staining and Sirius Red staining revealed that AMSC‐sEVs reduced fibrosis of colon tissues in CD mice. AMSC‐sEVs‐shMFGE8 promoted fibrosis of colon tissues in CD mice (Figure 7I–K). Furthermore, WB confirmed that AMSC‐sEVs significantly inhibited α‐SMA, COL1A1 and p‐p38 MAPK expression levels, which were reversed by MFGE8 knockdown (Figure 7L). Flow cytometry assay revealed that AMSC‐sEVs significantly reduced the proportion of F4/80^+^α‐SMA^+^ Mø and FoxP3^+^Treg, whilst shMFGE8 treatment led to an increase (Figure 7M,N). IF double‐labelling showed that AMSC‐sEVs treatment significantly decreased CD68^+^α‐SMA^+^ double‐positive cells and α‐SMA^+^CCL17^+^ cells, and shMFGE8 treatment led to significant up‐regulation of CD68^+^α‐SMA^+^ double‐positive cells and α‐SMA^+^CCL17^+^ cells (Figure 7O,P). All these results implied that AMSC‐sEVs delivered MFGE8 to inhibit MMT and intestinal fibrosis in CD mice.

## 4 Discussion

CD is a chronic non‐specific IBD that can involve the entire digestive tract [24]. In this work, we found that large numbers of Mø and Tregs were present in the stricturing intestinal tissues of patients with CD (illustrated by Figure 1). In vivo, increased infiltration of Mø‐derived myofibroblasts and Tregs was detected in colon tissues of CD mice (illustrated by Figure 2). Further studies found that Mø inoculation increased the proportion of MMT and Treg in CD mice (illustrated by Figure 3). TGF‐β1 induced MMT by activating the MAPK signalling pathway (illustrated by Figure 4). Mø differentiated into myofibroblasts and secreted CCL17, and then recruited Tregs by binding to the receptor CCR4 on the surface of Tregs (illustrated by Figure 5). Additionally, AMSC‐sEVs inhibited the MAPK signalling pathway through the delivery of MFGE8, thereby interfering with the MMT and ultimately ameliorating intestinal fibrosis in mice (illustrated by Figures 6 and 7).

Many different triggers can lead to the development of progressive fibrosis disease [25]. Regardless of the initiating trigger, a common feature of all fibrotic diseases is the activation of ECM‐producing myofibroblasts, a key mediator of fibrotic tissue remodelling [26]. The myofibroblast‐characteristically expressed gene α‐SMA is not expressed in quiescent myofibroblasts, making it unique in differentiated myofibroblasts. In this work, we found that the expression of α‐SMA was highly expressed in stricturing intestinal tissues of CD patients. It implied that there were a large number of activated myofibroblasts in the stricturing intestinal tissues. Additionally, the proportion of α‐SMA^+^CD68^+^ Mø (Mø‐derived myofibroblasts) was notably increased in stricturing intestinal tissues of CD patients. The same results were observed in colon tissues of CD mice. Multiple studies have shown that MMT is closely related to fibrotic diseases such as cardiac fibrosis, renal fibrosis, and pulmonary fibrosis [27]. Mø‐derived myofibroblasts gather in the areas of injury and inflammation, which contribute to ECM deposition and fibrosis [28]. Studies have shown that the TGF‐β1‐activated Smad3 signalling pathway induces MMT in renal fibrosis [29], whilst M2‐like Mø participate in MMT in pre‐retinal fibrosis [30]. However, the specific mechanism of action of MMT in CD has not yet been explored. In the present work, we confirmed that the proportions of Mø‐derived myofibroblasts were notably decreased in Mø‐depleted CD mice. Adoptive transfer of Mø promoted the MMT process, thereby alleviating fibrosis of colon tissues in CD mice.

Tregs are a subpopulation of CD4^+^ T cells with significant immunosuppressive effects [31]. It can inhibit the induction and proliferation of effector T cells, thereby regulating the immunity and maintaining immune balance in the organism. Tregs‐based therapy has been found to show better efficacy in systemic inflammatory and autoimmune diseases, including IBD and rheumatoid arthritis [32, 33]. The present work demonstrated that Tregs were abundant in CD mice. MMT cells secreted CCL17, and CCL17 interacted with the receptor CCR4 on the surface of Tregs to recruit Tregs. As inflammation continues, large amounts of TGF‐β secreted by Treg cells can activate the TGF‐β/Smad signalling pathway in intestinal mesenchymal cells [10, 11]. The activated TGF‐β/Smad signalling pathway is an important cause of the progression of intestinal fibrosis [34]. TGF‐β binds to TGF‐β receptors on the membrane surface of fibroblasts and phosphorylates downstream Smad2 and Smad3 [35]. Phosphorylated Smad2 and Smad3 form a transcriptional regulatory complex with Smad4, thereby leading to increased ECM secretion and ultimately the formation of intestinal fibrosis [36]. Thus, this work demonstrated that MMT cells recruited Tregs to the intestinal injury sites through the interaction between CCL17 and CCR4. With the accumulation of TGF‐β secreted by Tregs, a large number of fibroblasts in the intestine are activated. This may be a potential mechanism by which Tregs promote the progression of intestinal fibrosis.

AMSC‐sEVs, as a cell‐free therapeutic strategy, can regulate target cell function by delivering bioactive molecules such as proteins [16]. This study found that AMSC‐sEVs significantly inhibited the activation of the MAPK signalling pathway, disrupted the MMT process and reduced CCL17‐mediated Treg recruitment by delivering the multifunctional anti‐fibrotic protein MFGE8, ultimately improving intestinal fibrosis in the CD mouse model. Notably, knocking down MFGE8 significantly weakened the anti‐fibrotic effect of AMSC‐sEVs, further confirming MFGE8's central role in this process. Consistent with the findings of this study, Shi et al. [37] found that MFGE8 alleviates pulmonary fibrosis in acute lung injury by inhibiting the BMP/Smad1/5‐Smad4 pathway, whilst Xiong et al. [16] also confirmed in an intestinal fibrosis model that MFGE8 can regulate the Fak/Akt pathway to inhibit fibrosis progression. Additionally, studies have shown that encapsulated adipose‐derived mesenchymal stem cells can block myofibroblast activation by regulating the MFGE8/STAT3/Arg1 axis [38], further highlighting the potential of extracellular vesicles as drug delivery carriers. These findings collectively confirm the importance of MFGE8 in anti‐fibrotic therapy and provide a scientific basis for AMSC‐sEVs‐based CD treatment strategies. In the future, further investigation into the interactions between MFGE8 and other fibrosis signalling pathways, coupled with optimization of AMSC‐sEVs delivery efficiency, is expected to enhance their potential in CD therapy.

Although this study provides compelling evidence for the role of MMT in CD‐related fibrosis, there are some limitations. First, TNBS‐induced mouse models, although widely used, do not fully replicate the chronic and multifactorial nature of human CD. Future studies could validate these results in genetic CD models or patient‐derived organ tissues. Second, the therapeutic effects of AMSCs' sEVs were evaluated through short‐term interventions, and further evaluation of their long‐term safety and efficacy is needed. Additionally, whilst our current safety assessment of AMSC‐sEVs showed no short‐term adverse effects during the treatment period, a more comprehensive evaluation of their long‐term biosafety profile, including potential immunogenicity and tumorigenicity, is warranted. The biodistribution and optimal administration strategies of AMSC‐sEVs also require further investigation using advanced tracing techniques to provide stronger support for their clinical translation. Finally, the exact mechanism by which MFGE8 inhibits MAPK signalling remains unclear, and downstream targets need to be identified with the help of proteomics or single‐cell RNA sequencing.

## 5 Conclusion

In conclusion, this work demonstrated that TGF‐β1 activated the MAPK signalling pathway and promoted the MMT and secretion of CCL17 under CD‐associated intestinal inflammatory conditions. CCL17 promoted intestinal fibrosis by binding to the receptor CCR4 on the surface of Tregs and recruiting Tregs to intestinal injury sites. AMSC‐sEVs inhibited the MAPK signalling pathway through the delivery of MFGE8, thereby interfering with the MMT and ultimately ameliorating intestinal fibrosis.

## Author Contributions

**Minghao Xie:** conceptualization, formal analysis, project administration, methodology, writing – original draft preparation, writing – review and editing. **Qiang Liu:** formal analysis, project administration, methodology, software; **Zhizhong Xiong:** data curation, formal analysis, methodology, writing – original draft preparation, investigation. **Jian Li:** formal analysis, methodology, software, validation, visualisation. **Ruiri Jin:** formal analysis, investigation, software, data curation. **Lei Lian:** data curation, validation, visualisation. **Zhengrong Li:** resources, supervision, funding acquisition, writing – review and editing.

## Funding

This work was supported by Jiangxi Provincial Natural Science Foundation (Grant Nos. 20224BAB216022, 20232BAB206020) and National Natural Science Foundation of China (Grant No. 82400612).

## Ethics Statement

The protocols conformed to the Declaration of Helsinki and were approved by the ethical review committee of the Sixth Affiliated Hospital of Sun Yat‐sen University (2021ZSLYEC‐075). The animal experimental protocol was approved by the Ethical Committee of the First Affiliated Hospital, Jiangxi Medical College, Nanchang University (CDYFY‐IACUC‐202305QR045).

## Consent

All participants signed the informed consent.

## Conflicts of Interest

The authors declare no conflicts of interest.

## Data Availability

The datasets used and/or analyzed during the current study available from the corresponding author on reasonable request.
